# Design and Evaluation of a Pneumatic-Actuated Active Balance Board for Sitting Postural Control

**DOI:** 10.3390/s25237101

**Published:** 2025-11-21

**Authors:** Erkan Kaplanoglu, Max Jordon, Jeremy Bruce, Gazi Akgun

**Affiliations:** 1 College of Engineering and Computer Science, University of Tennessee at Chattanooga, Chattanooga, TN 37403, USA; gazi-akgun@utc.edu; 2 Physical Therapy, University of Tennessee at Chattanooga, Chattanooga, TN 37403, USA; max-jordon@utc.edu; 3 Department of Orthopaedic Surgery Chattanooga, University of Tennessee Health Science Center College of Medicine, Chattanooga, TN 37403, USA; jeremy.bruce@erlanger.org; 4 CUIP Research Institute, University of Tennessee at Chattanooga, Chattanooga, TN 37403, USA

**Keywords:** balance board, PAM, cLBP, rehabilitation

## Abstract

Chronic low back pain (cLBP) is a pervasive and debilitating condition that can result in motor control deficits and often leads to opioid dependence. Conventional rehabilitation approaches generally rely on internally driven tasks, which fail to capture adaptive motor responses to external perturbations. This study focuses on the design and evaluation of a pneumatic-actuated active balance board integrating pneumatic artificial muscles (PAMs), electromyography (EMG), and inertial measurement units (IMUs) to assess seated postural control responses. With PAM-powered perturbations, the balance board introduces controlled challenges to evaluate postural control dynamics and motor adaptation. EMG sensors monitor muscle activity in key postural muscles, while IMU systems track movement responses. The system was evaluated through an experimental trial with 15 healthy participants performing balance tasks on both a passive and active balance board. The active balance board’s effectiveness is assessed using signal processing techniques, including root mean square (RMS) analysis, Fast Fourier Transform (FFT), autoregressive (AR) modeling, and the Welch *t*-test. Experimental trials were conducted with healthy participants to establish baseline performance. Results demonstrate that the active balance board successfully induces adaptive motor responses, with higher EMG activation levels compared to passive boards. Frequency-domain analyses confirm significant differences in muscle activation patterns, supporting the hypothesis that external perturbations enhance postural control retraining. The pneumatic-actuated balance board presented in this study represents a novel approach to postural control assessment that may be applied in future rehabilitation studies involving individuals with cLBP, addressing the limitations of traditional methods. Future research will focus on clinical trials with cLBP patients to further evaluate its therapeutic efficacy and long-term benefits in rehabilitation.

## 1. Introduction

Chronic low back pain (cLBP) is one of the most serious health problems in the United States and around the world. It contributes to both disability rates and opioid use worldwide [1]. Despite advances in this area, the prevalence of cLBP is steadily rising, and more people are receiving opioid prescriptions to manage their discomfort [2]. Given the negative societal and economic consequences of cLBP, developing new approaches to assess and treat this condition is considered a critical priority by many international health organizations [3,4,5].

Given the absence of a clear link between anatomical variations in the spine and cLBP, motor control impairments and deficits in proprioception have been suggested as potential culprits [6,7]. Traditional assessments for motor control often focus on internally driven tasks, where the individual initiates the movement, but this overlooks reactive motor control, which requires individuals to respond to externally generated forces [8]. Additionally, research has indicated that individuals with cLBP exhibit deficits in motor planning, which may not be revealed through preplanned, internally generated movements of the lumbopelvic musculature [9]. Recent studies have emphasized that proprioceptive and neuromuscular training play a crucial role in improving postural balance and adaptive motor control across various populations, including older adults and athletes [10,11,12].

Recent studies have further elucidated the relationship between proprioception and postural control in individuals with cLBP. For instance, recent evidence has suggested that following a vibratory stimulus to the lower extremity, individuals with cLBP demonstrated poorer lumbar postural control and worse proprioception compared to asymptomatic controls [13]. Furthermore, recent meta-analytical evidence has emphasized the role of proprioceptive training in enhancing neuromuscular adaptation and postural stability across different populations, as reported by Ghai et al. [14] Additionally, a separate study investigated lower limb proprioception in individuals with cLBP found that, despite a lack of lower limb pain, proprioception was decreased compared to asymptomatic individuals [15]. Taken together, these studies suggest that cLBP not only results in proprioceptive deficits in the lumbar region, but also beyond.

Therefore, a key challenge to overcome in assessing motor control planning in individuals with cLBP is to isolate the contributions of the trunk musculature while simultaneously minimizing the errors that arise from the lower extremities. While posturographic measures in standing provide valuable insights for certain populations, they are of limited value in assessing trunk control in individuals with cLBP, as the hip, knee, and ankle joints often compensate for, or result from trunk deficiencies [16,17]. An alternative approach involves assessing motor control in a seated position on an unstable surface [18]. However, these approaches only assess motor control as it relates to an individual internally producing a movement. To date, there is a paucity of evidence evaluating reactive postural control in individuals with cLBP.

Recognizing these limitations, previous studies have investigated the influence of pain-related threat on motor behavior in individuals with nonspecific low back pain [19], emphasizing the need for objective assessment of reactive motor control. Building upon this need, active balance boards have emerged as promising tools for assessing and retraining postural stability by producing controlled perturbations that enhance balance control and sensorimotor adaptation [20,21]. Unlike their passive counterparts, active systems often integrate actuators and sensors to assess and retrain balance by eliciting adaptive motor responses. Sensor integration is highlighted as a crucial component in balance rehabilitation, enabling precise assessment and adaptive motor response training [22,23]. Moreover, balance assessment techniques based on geometric modeling and AI-driven feedback systems have increased the accuracy of tracking center of mass (CoM) and muscle activation patterns. Nevertheless, challenges remain in calibrating personalized interventions and optimizing real-time biofeedback mechanisms; therefore, further clinical trials and technological advancements are needed to maximize their efficacy in rehabilitation and sports performance [24].

Motivated by these research gaps and technological needs, the present study introduces a novel pneumatic-actuated balance board designed to provide dynamic, controlled perturbations for reactive postural assessment. We hypothesize that the pneumatic-actuated balance board will induce greater trunk muscle activation and more adaptive postural responses compared to a passive wobble board. The present study introduces a dynamic proprioceptive challenge to evaluate motor planning and control deficits in a controlled manner. The proposed system incorporates pneumatic artificial muscles (PAMs), also known as McKibben muscles, which mimic human muscle properties and offer advantages such as lightness, high force output, and a favorable power-to-weight ratio. The design principle was to create an adaptive, biomimetic, and safe rehabilitation platform. PAMs were chosen due to their high power-to-weight ratio, inherent compliance, and muscle-like contraction characteristics, making them well suited for generating dynamic perturbations. While electric actuators have good control, they typically do not have inherent compliance and safety for human-interactive applications, and hydraulic actuators can exert high forces but are heavy with potential leakage risks. PAMs, however, have compliance, lightness, and muscle-like actuation characteristics with inherent safety and efficacy in rehabilitation applications and thus suit our therapeutic objectives nicely.

PAMs have been widely adopted in soft robotics, prosthetics, and rehabilitation devices due to their structural similarity to human muscles. This technology allows for the generation of perturbation, enabling the assessment of adaptive motor control tasks and the dynamic training of lumbopelvic musculature [25,26,27].

EMG sensors were employed to objectively capture muscle activation patterns, and IMUs were employed to capture precise kinematic responses, enabling detailed assessment of dynamic motor control.

Electromyography (EMG) represents an essential tool for objective measurement of motor adaptation, allowing for real-time quantification of muscle activation and direct measurement of neuromuscular responses to dynamic perturbations. Through recordings from key postural muscles, EMG offers important insight into the nature of motor control deficits and adaptive processes and, as such, has become an essential element in rehabilitation outcome measurement.

The balance board proposed in this study uses electromyography (EMG) technology to monitor muscle activation in real time, focusing on the erector spinae, transverse abdominis, rectus abdominis, and external obliques. By capturing EMG signals, the device provides valuable data on symmetry and tone in postural muscle groups [23]. EMG measurements have been well established in low back pain research, demonstrating their utility in evaluating muscle activation patterns and assessing the effectiveness of interventions. For instance, Ahern et al. [28], showed that individuals with LBP exhibit reduced EMG readings during dynamic movements compared to healthy individuals, while Kohler [29] et al. utilized EMG signals to evaluate muscle activation in lifting applications. Therefore, the purpose of this study is to describe the creation of a novel, dynamic balance board that measures reactive postural control while measuring EMG from key muscle groups. The current study focuses on healthy participants to establish a baseline reference before extending to clinical populations with impaired motor control.

## 2. Materials and Methods

### 2.1. Experimental Protocol

In this study, to examine the performance of the proposed pneumatic-based balance board, EMG activity on the Pneumatic-Controlled Balance Board (PCBB) was compared with the Traditional Passive Balance Board (TBB, Yes4All Wobble Board) during seated balance tasks. MVIC values for the EO and ES muscles were obtained using validated procedures. Participants then performed five repetitions of pelvic tilts in the directions of 0°, 60°, 120°, 180°, 240°, and 300° while seated on the TBB. The TBB was fixed to a platform to provide the same sitting height as the pneumatic board, thus creating a similar stance. Following this step, participants were asked to move to the PCBB and stand upright while the board moved under them with the same parameters.

Fifteen healthy adults (mean age: 23 ± 3 years; seven females, eight males) participated in this study. Inclusion criteria included the absence of musculoskeletal or neurological disorders. Exclusion criteria included a history of low back pain, spinal surgery, or balance impairments. All participants were asymptomatic at the time of testing. The study was approved by the Institutional Review Board of the University of Tennessee at Chattanooga (FWA00004149, Protocol #21-028), and written informed consent was obtained from each participant prior to data collection.

EMG electrodes were bilaterally placed on the erector spinae (ES) and external obliques (EOs) following SENIAM guidelines [30,31,32]. EMG signals were filtered between 20 and 450 Hz using a band-pass filter, rectified, and smoothed using RMS envelopes. IMUs were centrally attached to the board and participant trunk for accurate recording. IMU data were low-pass filtered at 10 Hz. Both data streams were synchronized using a timestamped data acquisition protocol. Movement artifacts were minimized by precise fixation of the electrodes and visually verified prior to analysis.

The evaluation of the balance board was performed using signal processing techniques, specifically root mean square (RMS) analysis and autoregressive (AR) modeling, to assess muscle activation stability and reliability. The first evaluation step involved examining the stability of the collected EMG data. A comparison of mean versus RMS values was conducted to detect any anomalies or inconsistencies. The results indicated minimal deviations between the mean and RMS values, confirming that the collected data were stable and free of artifacts.

The second evaluation step compared EMG activation levels during active balance board trials with participants’ MVIC values. The analysis revealed that muscle 1 reached approximately 95% of its MVIC, while muscle 3 achieved 90% of its MVIC. These findings confirmed that the active balance board condition elicited significantly higher muscle activation than passive exercises, suggesting that externally generated perturbations play a key role in enhancing postural control training. The order of testing (active vs. passive board) was randomized across participants to minimize order effects.

To further evaluate the system, Fast Fourier Transform (FFT) analysis was applied to the EMG signals. This analysis revealed distinct shifts in dominant frequency ranges during active balance board trials compared to passive conditions, indicating that externally generated perturbations influenced neuromuscular activation patterns. Higher frequency components in the active condition suggest increased muscle engagement and adaptation to dynamic balance challenges.

Additionally, RMS and AR modeling are employed to quantify the differences in signal characteristics between active and passive conditions. The RMS values indicated greater muscle activation during active trials, supporting the hypothesis that controlled perturbations improve neuromuscular responses. AR modeling provided further evidence by demonstrating differences in signal complexity, suggesting that the active condition induced more adaptive motor responses.

The combination of these evaluation techniques confirmed that the pneumatic-actuated balance board effectively induced adaptive motor responses. Higher EMG activation levels, distinct frequency-domain muscle activation patterns, and robust RMS and AR comparisons provided strong evidence that externally generated perturbations enhance postural control retraining. These findings highlight the potential of the active balance board as a valuable tool for rehabilitation and motor control assessment.

### 2.2. Pneumatic-Controlled Active Balance Board Design

**Figure 1:**
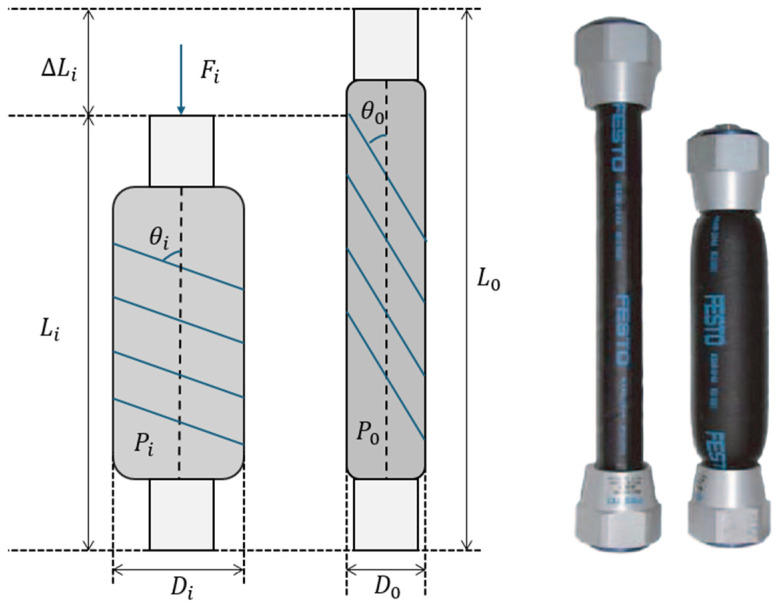
Pneumatic artificial muscle structure.

PAMs are soft actuators that use compressed air for their mechanical movement. Basically, they are made up of a flexible membrane in the shape of a cylinder with a braided shell. Upon entrance of compressed air, the membrane expands radially and contracts axially. This results in the creation of a pulling force. As air exits, this membrane elongates along its axis, as shown in Figure 1. This mechanism enables PAMs to realize linear and unidirectional movements, which makes them favorable for robotic or assistive use [25,33,34].

McKibben PAMs operate based on the interaction between internal pressure and structural deformation [35]. The muscle is made of a cylindrical membrane covered with braided fibers. Under internal pressure P, the membrane expands radially ($D_{i}>D_{0}$) while contracting axially ($L_{i}<L_{0}$). This deformation is geometrically related to the initial and final braid angles ($\theta_{0},\theta_{i}$), which influence force generation [34,36,37]. Based on the principle of conservation of energy, the force exerted by a PAM can be modeled to lead to the fundamental equation.

Using the Chou–Hannaford [36] model, the pulling force produced by PAM when pressure is applied can be written as Equation (1).

(1) $$F_{i}=\left(\frac{1}{4}\right)P_{i}A_{i}\left(3\;\cos^{2}{(\theta }_{i})-1\right)$$

where $F_{i}$ is the produced pulling force, $P_{i}$ is pressure, $A_{i}$ is the cross-sectional area of PAM, and θ is the angle between a braided thread, as depicted in Figure 1. We can describe the height of the PAMs and the braid angles using the Chou–Hannaford approach, as shown in Equation (2) [36].

(2) $$\theta_{i}=\cos^{-1}\left(\frac{L_{i}}{b}\right)$$

where $L_{i}$ is the length of the cylinder in meters and b is the whole fiber length in meters. Basically, a change in displacement depends on the forces exerted and applied to the system. Thus, we can easily calculate the contraction rate of the PAM with Equation (3).

(3) $$\lambda =\frac{L_{i}}{L_{0}}=\cos^{2}\theta +\frac{F_{ext}}{PA}$$

where λ is the contraction rate representing the relative change in PAM length as a function of applied external force and internal pressure. This ratio defines the PAM’s deformation, which is crucial for controlling platform movement, and it depends on the braid angle θ.

**Figure 2:**
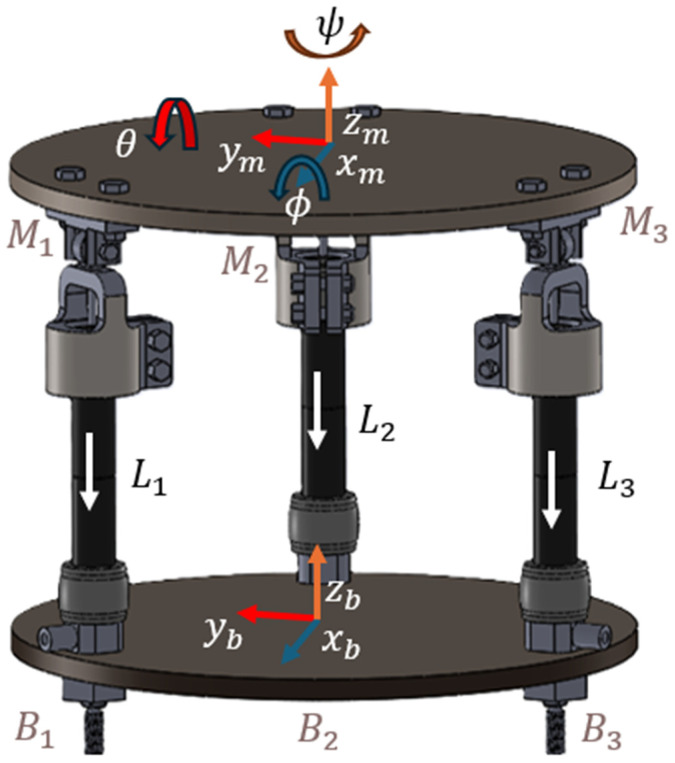
Structure of the PAM-actuated balance board. Colored arrows indicate the coordinate axes: red (x), blue (y), and orange (z). Rotation angles φ, θ, and ψ correspond to roll, pitch, and yaw of the moving platform.

As shown in Figure 2, the balance board design proposed in this paper consists of three PAMs connected at the apex points of an equilateral triangle, forming the base and moving platform.

For the system, each link length is given as $L_{1},L_{2},{and\;L}_{3}$. The moving platform position and orientation [x,y,z,ϕ,θ,ψ] can be found with forward kinematics. Here, (ϕ,θ,ψ) are Euler angles like for rotation about the *x*-axis (Roll ϕ), *y*-axis (Pitch θ), and *z*-axis $\left(Yaw\;\psi \right)$, respectively.

If the desired position and orientation of the moving platform is given, the base connection joints ($B_{i},i=1,\;2,\;3$) are defined in Equation (4).

(4) $$B_{i}=\left[\begin{array}{ccc} Rcos\left(0\right) & Rsin\left(0\right) & 0\\ Rcos\left(2\pi /3\right) & Rsin\left(2\pi /3\right) & 0\\ Rcos\left(4\pi /3\right) & Rsin\left(4\pi /3\right) & 0 \end{array}\right]$$

where R is the radius of the base platform. For the moving platform, we can calculate the connection points as follows:

(5) $$M_{i}=\left[\begin{array}{ccc} rcos\left(0\right) & rsin\left(0\right) & h\\ rcos\left(2\pi /3\right) & rsin\left(2\pi /3\right) & h\\ rcos\left(4\pi /3\right) & rsin\left(4\pi /3\right) & h \end{array}\right]$$

where r is the radius of the moving platform. The points $M_{i}\;,(\;i=1,\;2,\;3)$ are depicted with transformation matrices based on the position and orientation [x,y,z,ϕ,θ,ψ] of the moving platform.

(6) $$M_{i}^{\prime }=R\left(\phi ,\theta ,\psi \right).M_{i}+\left[x_{m},y_{m},z_{m}\right]$$

where R(ϕ,θ,ψ) is the transformation matrix depending on the Euler angles. The length of each link can be calculated as follows:

(7) $$L_{i}=\left\|M_{i}^{\prime }-B_{i}\right\|\left(i=1,2,3\right)$$

The constraint equations for each link are as follows:

(8) $${\left(x_{m}+R_{m}\;M_{i}^{0}-B_{i}\right)}^{T}\left(x_{m}+R_{m}M_{i}^{0}-B_{i}\right)=L_{i}^{2}$$

**Figure 3:**
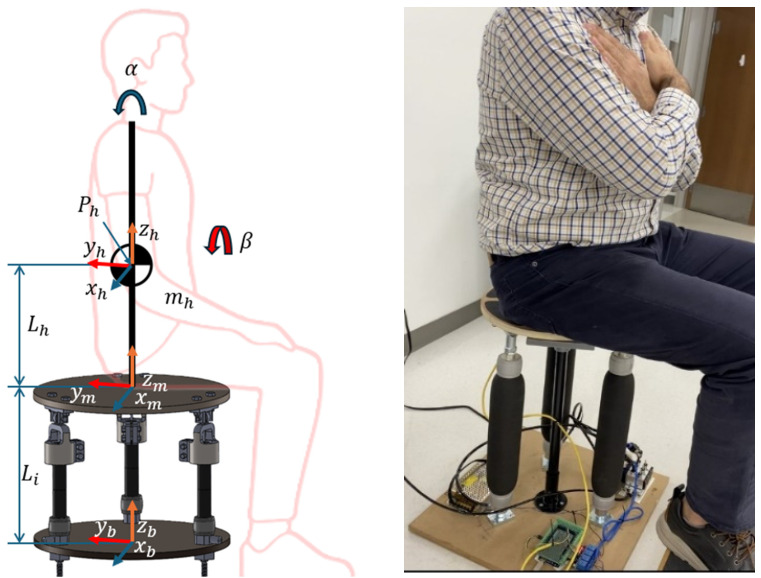
Experimental setup showing the articulated Stewart-platform–based balance trainer and its human–platform coordinate system. The schematic (left) illustrates the platform coordinates $\left(x_{b},\;y_{b},\;z_{b}\;and\;x_{m},\;y_{m},\;z_{m}\right)$, the upper-body mass m_h, and angular motions α (forward–backward) and β (side-to-side). The photo (right) shows a participant seated on the device during testing.

To obtain the equations of motion, including the effects of Euler angles, we use Lagrange’s formulation. The kinetic energy must account for both translational and rotational motion. If we consider adding a sitting human to the system, as seen in Figure 3, we can consider the human as an inverted pendulum connected to the center of the moving platform’s pivot point.

The endpoint position of the pendulum in the upper platform frame is as follows:

(9) $$P_{h}=\left[\begin{array}{c} x_{m}\\ y_{m}\\ z_{m} \end{array}\right]+R_{m}\left[\begin{array}{c} L_{h}sin\alpha \\ L_{h}sin\beta \\ -L_{h}cos\alpha cos\beta \end{array}\right]$$

where $R_{m}$ is the rotation matrix of the upper platform, and the pendulum’s motion is represented by the angles α and β. The velocity of the pendulum’s tip can be computed by differentiating $P_{h}$.

(10) $$\dot{P_{h}}={\dot{x}}_{m}+{\dot{R}}_{m}\left[\begin{array}{c} L_{h}sin\alpha \\ L_{h}sin\beta \\ -L_{h}cos\alpha cos\beta \end{array}\right]+R_{m}\left[\begin{array}{c} L_{h}cos\alpha \dot{\alpha }\\ L_{h}cos\beta \dot{\beta }\\ L_{h}\left(sin\alpha cos\beta \dot{\alpha }+sin\beta cos\alpha \dot{\beta }\right) \end{array}\right]$$

The total energy consists of, $T_{m}=\frac{1}{2}M_{m}\left({\dot{x}}_{m}^{2}+{\dot{y}}_{m}^{2}+{\dot{z}}_{m}^{2}\right)+\frac{1}{2}\omega_{m}^{T}I_{m}\omega_{m}$ for moving the upper platform, and $T_{h}=\frac{1}{2}m_{h}\left({\dot{P}}_{p_{x}}^{2}+{\dot{P}}_{p_{y}}^{2}+{\dot{P}}_{p_{z}}^{2}\right)+\frac{1}{2}I_{h}\left({\dot{\alpha }}^{2}+{\dot{\beta }}^{2}\right)$ for the inverted pendulum model for human involvement in the system, where $I_{h}$ is the moment of inertia of the pendulum. The potential energy consists of the following:

(11) $$V_{m}=M_{m}gz_{m}$$

(12) $$V_{h}=m_{h}g(z_{m}-I_{h}cos\alpha cos\beta )$$

(13) $$V_{s}=\sum_{i=1}^{3}\frac{1}{2}k_{i}{\left(L_{i}-L_{i_{0}}\right)}^{2}$$

where $V_{m},\;V_{h},$ and $V_{s}$ are potential energies of the balance board, the inverted pendulum, and the elastic forces from the platform legs, respectively.

To extract the motion equations, we can solve the Lagrange equation for α and β as shown in Equation (14) and Equation (15).

(14) $$\frac{d}{dt}\left(\frac{\partial L}{\partial \dot{\alpha }}\right)-\frac{\partial L}{\partial \alpha }=\tau_{\alpha }$$

(15) $$\frac{d}{dt}\left(\frac{\partial L}{\partial \dot{\beta }}\right)-\frac{\partial L}{\partial \beta }=\tau_{\beta }$$

**Figure 4:**
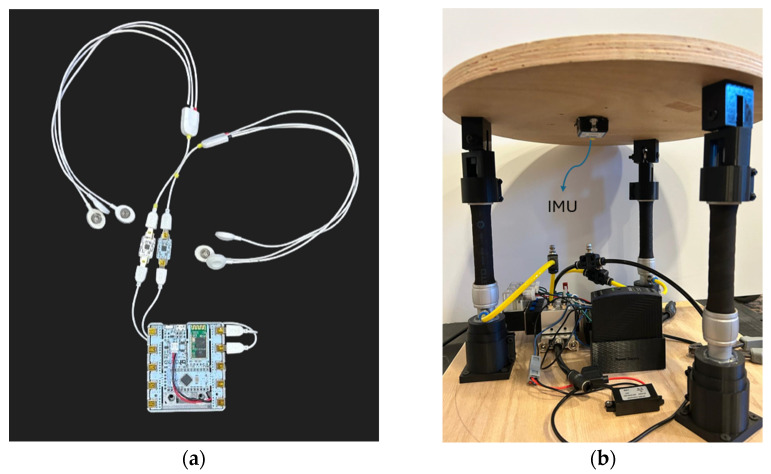
Sensor integration. (**a**) on the left picture (EMG sensor + board); (**b**) on the right picture (IMU under platform).

To measure how effective the board is at stimulating the abdomen and lower back areas, two subsystems were implemented. The first being an EMG data acquisition subsystem, this is performed by using a wearable device and PCB board designed to collect EMG readings among other bio signals. The wearable EMG system (Bitalino) is shown in Figure 4.

**Figure 5:**
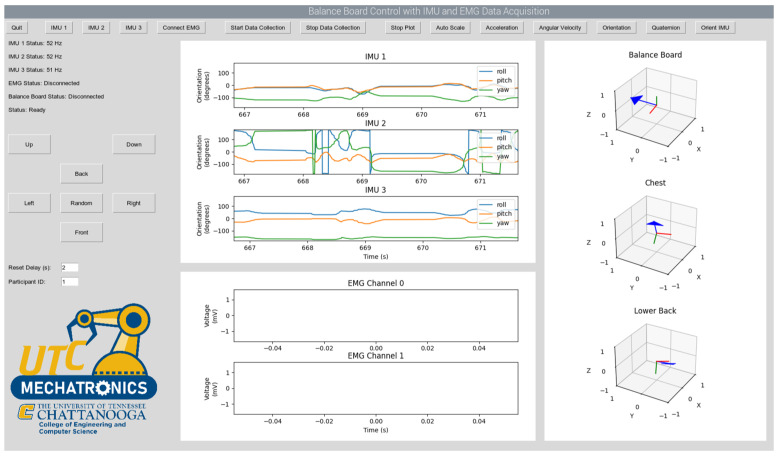
Graphic user interface.

The second subsystem needed is an inertial measurement unit (IMU), also shown in Figure 4, which is connected to the microcontroller. The purpose of the IMU is to demonstrate that EMG activity corresponded with the board’s movement. Analog data received from the IMU sensors and EMG sensors will be converted to digital data with a data acquisition card and transferred to a computer. The system will have an electronic board that drives the valves that activate the pneumatic muscles. The kinematic data recorded from the participants will be analyzed using a GUI. A graphic user interface (GUI) is created for controlling and collecting data. The GUI was developed in MATLAB App Designer (R2023b, Mathworks, Natick, MA, USA) and Sketch 3.7.2, Sektch B.V., The Hague, The Netherlands) for both computers and mobile phones. Figure 5 shows the GUI.

The graphical user interface (GUI), shown in the screenshot in Figure 5, was developed to monitor data flow and operation. The balance board interface collects data from the IMU and EMG sensors at sampling frequencies of 10 Hz and 1000 Hz, respectively. A control algorithm runs predefined scenarios, generating control signals for the pneumatic actuators and sending them to the hardware. All data flow read from the sensors during operation are displayed and recorded on the interface. Developed in Python (version 3.10, Python Software Foundation, Wilmington, DE, USA) programming language, the GUI offers a flexible structure for sensor integration.

To maintain system speed, the software runs the user interface, IMU data collection, EMG data collection, and drawing operations in separate threads. The project uses WitMotion WT901BLECL IMU devices and BITalino (r)evolution Board EMG sensors. Wireless serial communication between the computer and the microcontroller is provided via Bluetooth. The software displays both kinematic data and EMG channels in real time.

## 3. Results

### 3.1. Simulation Results

**Figure 6:**
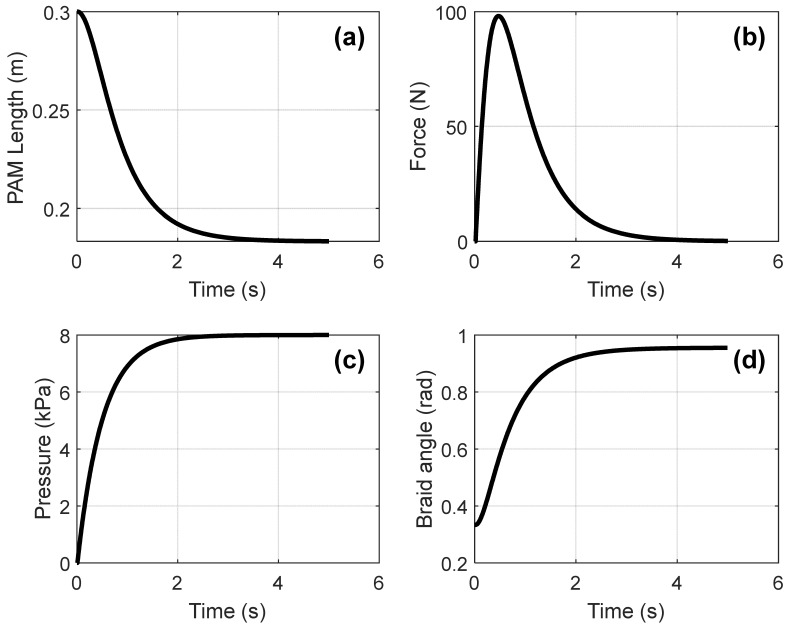
Response of a single PAM under pressure. (**a**) Variation of PAM length over time; (**b**) Force response generated by the PAM; (**c**) Pressure evolution inside the actuator; (**d**) Braid angle change during activation.

When we put a mass on the PAM and pressure is gradually increased to 10 bar (1000 kPa) over 2 s, the dynamic behavior of the system can be observed, as shown in Figure 6. The length of the PAM decreases from 0.3 m to 0.15 m, and the force produced by the PAM changes over time, as shown in Figure 6. When the braid angle reaches the mechanical limit of the PAM, it stops moving.

**Figure 7:**
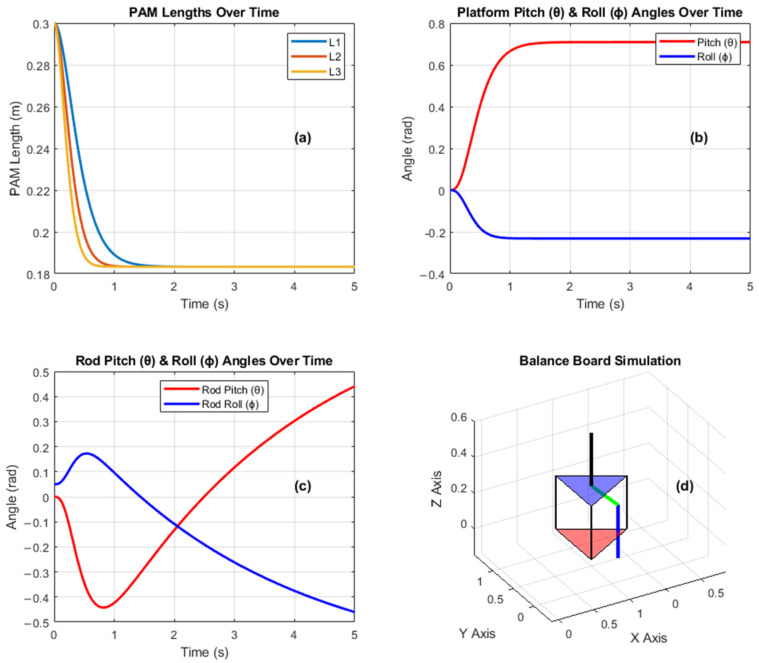
Simulation results for the balance board model with P=[2.5,5,8] in bar. (**a**) PAM lengths L_1 L_2 L_3 over time showing rapid initial contraction and convergence to steady-state; (**b**) Platform pitch θ and roll ϕ angles, indicating fast pitch stabilization and minimal roll deviation; (**c**) Rod pitch θ and roll ϕ responses, illustrating counteracting trends and dynamic coupling effects; (**d**) 3D balance board simulation depicting the platform configuration and rod orientation at the end of motion.

The whole simulation results for the balance board can be seen in Figure 7. For this simulation, P=[2.5,5,8] pressures were applied to all PAMs. As seen in Figure 7a, the of each PAM reduces 0.3 m to ~0.18 m when the platform’s Euler angles, like Pitch (θ) and Roll (ϕ), change. For this case, the Euler angles representing the inverted pendulum with the human sitting on the platform also change as seen Figure 7d. As seen in Figure 7c, while the Pitch (θ) angle is increases to 0.5, the Roll (ϕ) angle decreases to ~−0.5 rad degrees.

**Figure 8:**
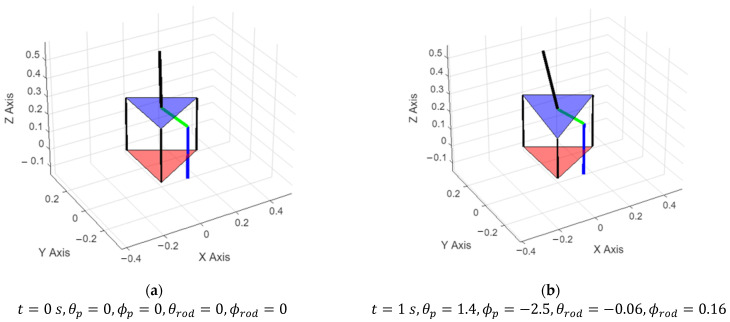

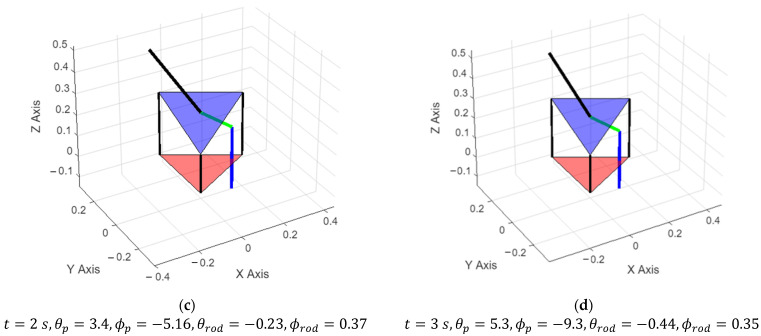
Simulation results for the balance board model with *P* = [0, 0, 8] in bar. (**a**) Balance board position on *t* = 0 s; (**b**) Balance board position on *t* = 1 s; (**c**) Balance board position on *t* = 2 s; (**d**) Balance board position on *t* = 3 s.

To simulate the effect of different scenarios, we can apply different pressures to the PAMs and analyze the outcomes. As seen in Figure 8, if the pressure matrix is [0, 0, 8], then just the PAM goes down while the rest of them keep their position at 0.3 in meters. When pressure is applied to the system, just one PAM length changes, and the moving platform follows the PAMs. As seen in Figure 8, humans sitting on the balance board reflect this action as changes in their Euler angles.

### 3.2. Evaluation Experiment Results

The first thing to look at when analyzing the data is the stability of the data. Table 1 shows the mean versus root-mean-squared (RMS) of all data collected for a single participant. This comparison uses unfiltered data; however, it is a preliminary evaluation step to look for abnormalities in the collected data. Looking at Table 1, the difference between the mean and RMS are small, indicating that the data has no abnormalities.

**Figure 9:**
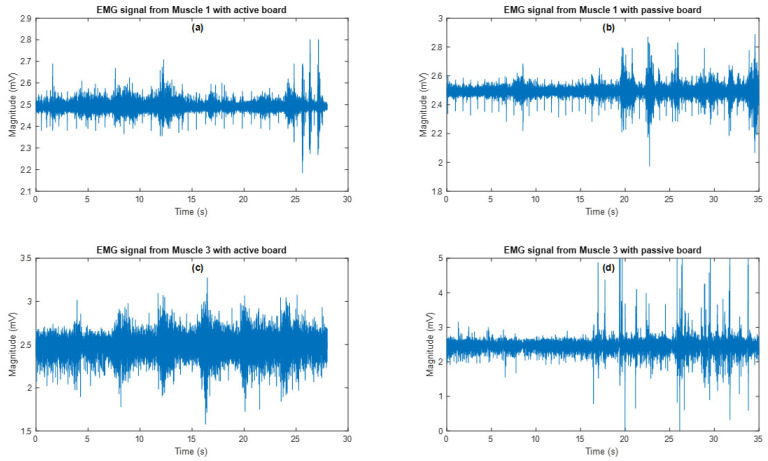
Components of EMG signals in the frequency domain. (**a**) EMG signal from Muscle 1 with active board; (**b**) EMG signal from Muscle 1 with passive board; (**c**) EMG signal from Muscle 3 with active board; (**d**) EMG signal from Muscle 3 with passive board.

**Figure 10:**
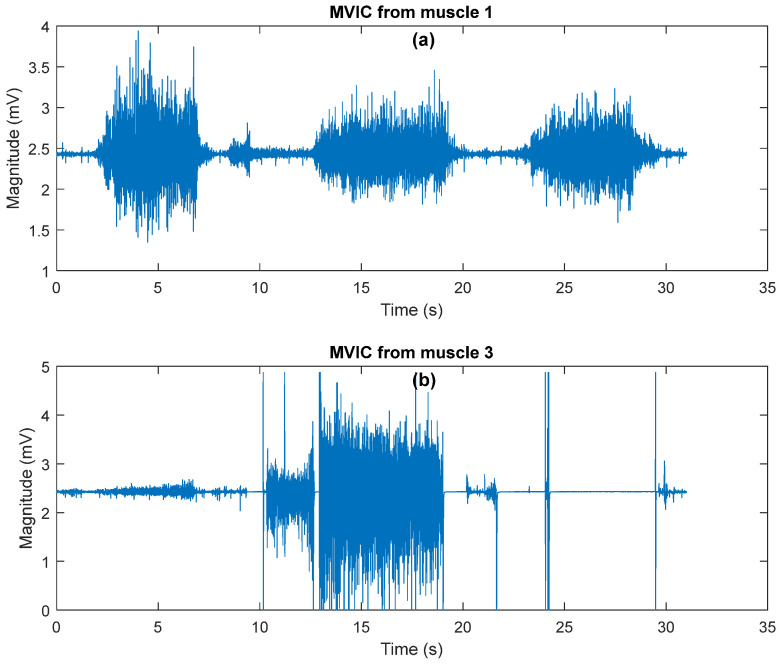
MVIC from muscles. (**a**) MVIC from muscle 1; (**b**) MVIC from muscle 3.

The results from Table 1 are similar for all participants, indicating that all data is free of anomalies. The second thing used to validate the design is comparing the active balance board to Maximum Volitional Isometric Activity (MVIC) values. The graphs in Figure 9 show the EMG signals measured in these experiments. When compared to MVIC shown Figure 10, it is discovered that muscle 1 reached 95 percent of its MVIC in both experiments, while muscle 3 reached 90 percent of its MVIC in both experiments.

**Figure 11:**
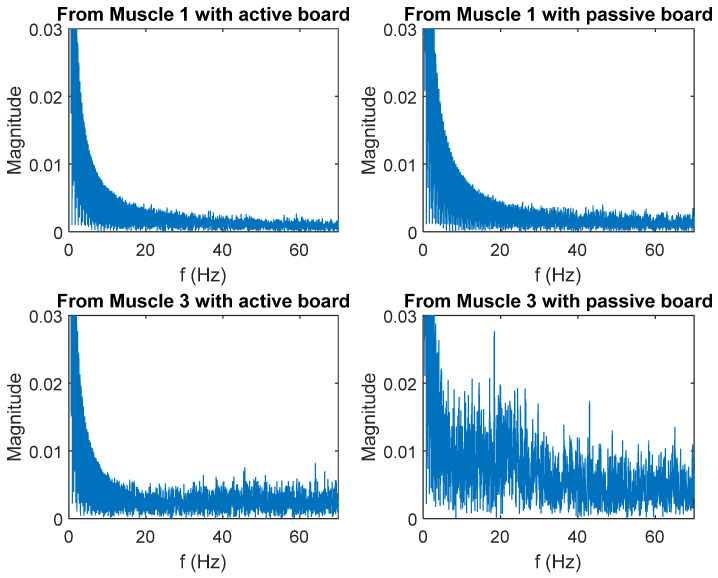
Components of EMG signals in the frequency domain muscle 1 = left erector spinae, muscle 2 = right erector spinae, and muscle 3 = external oblique.

The frequency components of the EMG signals received from each muscle are examined using the Fast Fourier Transform (FFT) process. Figure 11 depicts the results.

The weighted average value of the amplitude and frequency vectors is calculated as in Equation (16) to calculate the mean frequency value for each experiment.

(16) $$wf=\frac{FxMag}{\sum_{i=1}^{n}Mag}$$

Table 2 shows values of the weighted frequency calculated from EMG signals from muscle 1 and muscle 3 with active and passive exercises.

In the time domain, two different features have been calculated for comparison: the root mean square (RMS), shown in Equation (17), and the *n*-th order autoregression vectors, shown in Equation (18).

(17) $$rms\text{\_}feat=\sqrt{\frac{1}{n}\sum_{i=1}^{n}x_{i}}$$

(18) $${ar}_{t}=\left(1-\phi_{1}B-\phi_{2}B^{2}\right)X_{t}$$

**Figure 12:**
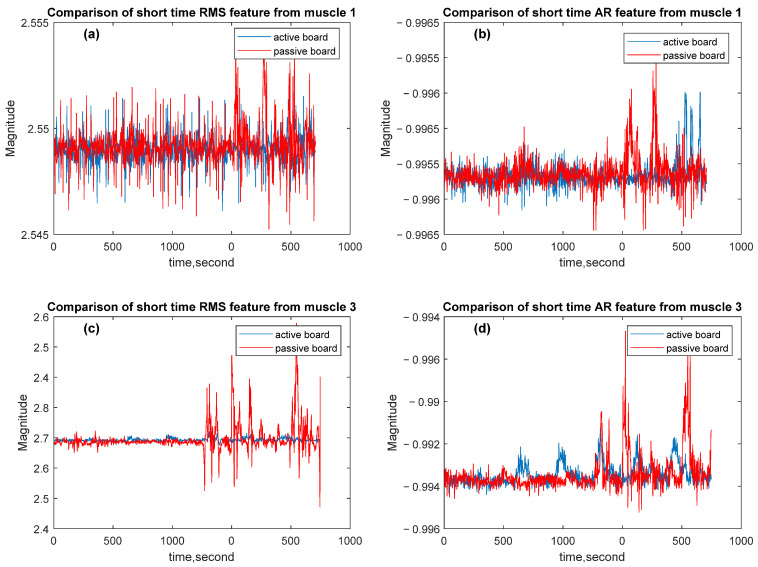
Comparative graphs of RMS and AR features of the EMG signals. (**a**) RMS feature for muscle 1; (**b**) AR feature for muscle 1; (**c**) RMS feature for muscle 3; (**d**) AR feature for muscle 3.

The signal fragments are obtained by dividing the signal into specific and short-time windows in the time axis. Calculated features are shown in Figure 12.

The RMS and AR values are calculated as features. For comparison of these features, mean values are calculated for all signals, and the mean percentage of absolute errors is used to compare these values. For muscle 1, the mean percentage error between the RMS values of the active and passive experiments is calculated as ${7.58\times 10}^{-5}$. Other values are shown in Table 3.

To address how much percent of MVIC muscles contracted during passive or active balance board trials, the % MVIC ($\frac{\max\left(RMS\left(t\right)\right)}{RMS_{MVIC}}\times \;100$) and the total muscle activity area under the curve (AUC) $(\int_{0}^{T}MVIC\left(t\right)dt)\;$ are calculated. For the relative duration during which the muscle activation level exceeds a meaningful (%10 of MVIC) threshold, the active time ratio (> 10% MVIC) is calculated as $t\left(\left|\%MVIC(t)\right|>10\right)$/T. Together these metrics characterize the muscle response in both magnitude and duration, while AUC integrates both aspects to describe total neuromuscular effort. By combining these measure with statistical analysis, the study provides a comprehensive picture of how externally driven perturbations influence muscle activation strategies and postural control.

The findings of the analysis are shown in Table 4. Peak % MVIC represents the highest normalized EMG amplitude reached by the muscle during each trial and represents the maximum intensity of voluntary or reflexive muscle contraction. Peak values were significantly higher in the active condition for most participants. For example, P5: 53.5% (A) vs. 10.6% (P), P10: 54.5% (A) vs. 19.6% (P), and P11: 62.6% (A) vs. 22.9% (P). These differences suggest that external perturbations generated by the pneumatic system require faster and stronger muscle responses to maintain balance. In other words, the active platform increased motor unit recruitment in the muscles, leading to more dynamic muscle contractions compared to the passive condition.

The area under the normalized EMG curve (AUC) quantifies the total neuromuscular energy output generated by the muscle during the trial. For most participants (P4, P10, P11, P14), AUC values were higher in the active trials, indicating increased total muscle workload in response to sustained perturbations.

However, some individuals (P1, P9, P15) had higher AUC values in the passive condition. This may be due to individual compensation strategies, some individuals may have maintained balance by maintaining low muscle activity for extended periods, even in the absence of active perturbations. In general, higher AUC values in the active condition indicate that pneumatic perturbations require sustained neuromuscular engagement and dynamic stabilization, thus supporting the hypothesis that externally driven balance challenges increase muscle recruitment and postural control.

This metric represents the ratio of the time during which the EMG amplitude exceeds the physiologically significant 10% MVIC threshold to the total time. Longer active time proportions (e.g., P5, P8, P10, P11, P13) in active conditions indicate that the muscles remain active for a greater portion of the task. This suggests an increased need for co-contraction and consistent muscle activation to maintain balance.

The findings support the notion that active balance perturbations promote sustained motor engagement and may facilitate neuroplastic adaptations through repetitive sensorimotor feedback.

## 4. Discussion

This study involves a cohort of 15 healthy, college-aged participants without a history of back pain, completing exercises designed to validate the device’s effectiveness. The EMG-responsive balance board offers a unique approach as a potential tool for addressing cLBP by providing externally generated perturbations in future rehabilitation studies, which challenge individuals in a controlled and measurable manner. Additionally, it supports physical therapists in applying targeted interventions, potentially improving outcomes for patients with cLBP.

The results obtained are consistent with proprioceptive and motor adaptation studies [38]. Specifically, in terms of EMG activity and postural adaptation, the active and passive trials conducted on the balance board demonstrate alignment with previously reported findings on proprioceptive feedback mechanisms and neuromuscular adaptation processes [39]. Similar improvements in postural control and neuromuscular coordination have been reported following proprioceptive and balance-based training interventions. For example, Concha-Cisternas et al. [10] demonstrated that neuromuscular training enhances balance and muscle activation in older adults, while Antohe et al. [11] found that proprioceptive exercises improved stability in athletes with chronic ankle instability. Zemková et al. [12] also described sport-specific neuromuscular adaptations that align with the increased EMG activity observed in our active balance board trials. In particular, the increase in EMG activity during active balance control corresponds to muscle activation patterns observed in previous motor learning and adaptation studies.

While the study presents promising findings, there are certain aspects that could be further refined. Since the study was conducted with healthy individuals, clinical trials involving participants with motor control impairments would be necessary to fully validate the effectiveness of the device for rehabilitation purposes. Additionally, optimizing the response time and pressure adjustments of the pneumatic system could enhance the board’s ability to provide more precise and controlled movements. Another consideration is the sample size; while the findings are valuable, a larger participant pool would strengthen the generalizability of the results.

The results obtained in the study demonstrate the system’s impact on balance and muscle activation. However, the participants were healthy and young, leaving future research to assess the validity of these findings for different age groups or clinical patients. Furthermore, the small delays observed in the pneumatic system’s response time could be improved with faster control algorithms. Larger, clinically based studies will better demonstrate the system’s true impact in rehabilitation practices. Future improvements could focus on integrating adaptive control systems to achieve more precise regulation of the PAMs through feedback-driven algorithms. Additionally, incorporating machine learning techniques into balance and postural control analysis could enable personalized adaptation models and training programs tailored to individual users. Enhancing motion tracking with advanced IMU and force sensors could further improve the system’s adaptability, allowing for a more customized experience based on user-specific needs.

## 5. Conclusions

This study develops a pneumatic-actuated active balance board designed for the assessment and retraining of postural control in patients with cLBP, integrating pneumatic artificial muscles (PAMs), electromyography (EMG), and inertial measurement units (IMUs). It introduces controlled perturbations that induce adaptive motor responses, addressing a well-known limitation of traditional rehabilitation exercises, which primarily rely on internally driven tasks. Experimental evaluation with 15 healthy participants demonstrated that the active balance board induced higher EMG activation levels and distinct frequency-domain muscle activation patterns, confirming its potential to enhance postural control retraining.

By integrating advanced sensor technology and open-source software, this project contributes to the broader landscape of rehabilitation technologies, offering a novel tool for addressing motor control deficits in individuals with cLBP. The dynamic EMG-responsive balance board has the potential to improve patient outcomes, reduce opioid dependency, and enhance the overall effectiveness of rehabilitation practices for chronic low back pain.

The findings suggest that externally generated perturbations play a crucial role in improving motor adaptation, which can inform the design of more effective rehabilitation strategies for patients with cLBP. The system further enhances objectivity by providing real-time data on muscle activation and movement responses, supporting various clinical and therapeutic applications.

## 6. Patents

Kaplanoglu, E. (15 April 2025). Athletic balance control platform with pneumatic actuation (U.S. Patent No. USD 1,071,046 S1). U.S. Patent and Trademark Office [40].

## Figures and Tables

**Table 1:** Comparison of mean and RMS of a participant’s raw data.

Trial Name	Channel	Mean	RMS	Difference
MVIC	1	509.7	509.9	0.2
	2	509	509.1	0.1
Passive	1	509.6	509.7	0.1
	2	508.9	508.9	0
Active 1	1	509.6	509.8	0.2
	2	508.6	508.6	0
Active 2	1	509.7	509.7	0
	2	508.7	508.7	0
Active 3	1	509.7	509.7	0
	2	508.7	508.7	0
Active 4	1	509.6	509.7	0.1
	2	508.7	508.8	0.1
Active 5	1	509.7	509.7	0
	2	508.7	508.8	0.1

RMS: root mean square and MVIC: Maximum Volitional Isometric.

**Table 2:** Weighted frequencies of EMG signals.

	Active Board	Passive Board
From Muscle 1	55.56 Hz	64.85 Hz
From Muscle 3	106.58 Hz	106.52 Hz

**Table 3:** Mean percentage absolute error of features calculated.

	MPE of RMS Values	MPE of AR Vectors
Muscle 1	$7.5856\times 10^{-5}$	$-1.3072\times 10^{-5}$
Muscle 3	0.0016	$-7.6061\times 10^{-5}$

MPE: max percentage error.

**Table 4:** Comparison of all data collected from participants.

	Peak % MVIC		AUC (%MVIC.s)		Active Time Ratio (> 10%MVIC)		Welch *t*-Test	*p* Value
	A	P	A	P	A	P	Between A and P	
P1	24.60	5.92	37.11	55.96	0	0	−9.90	*p* < 0.0001
P2	23.10	32	19.52	10.33	0	0	47.77	*p* < 0.0001
P3	54.26	24.41	33.01	38.62	0	0	−5.4	*p* < 0.0001
P4	43.54	21.24	62.51	45.25	0	0	35.25	*p* < 0.0001
P5	53.50	10.57	25.32	31.88	0.0076	0.0077	−16.46	*p* < 0.0001
P6	51.71	4.21	25.14	21.43	0.0072	0	11.54	*p* < 0.0001
P7	48.55	9.79	24.79	30.07	0	0	−14.01	*p* < 0.0001
P8	55.82	20.07	24.34	30.00	0.0075	0.047	−12.14	*p* < 0.0001
P9	55.33	27.54	38.99	76.79	0.0074	0.2169	−56	*p* < 0.0001
P10	54.49	19.60	41.50	11.50	0.0072	0.0880	−46	*p* < 0.0001
P11	62.63	22.86	66.20	47.80	0.0727	0.1210	31.44	*p* < 0.0001
P12	26.6	9.8	76.2	19.10	0	0	125.05	*p* < 0.0001
P13	54.07	19.36	45.05	62.71	0.0175	0.0976	−37.39	*p* < 0.0001
P14	10.17	1.40	65.44	56.11	0	0	13.75	*p* < 0.0001
P15	15.25	7.54	45.40	62.52	0	0	42.51	*p* < 0.0001

## Data Availability

The original contributions presented in this study are included in the article material. Further inquiries can be directed to the corresponding author.

## Abbreviations

The following abbreviations are used in this manuscript:

cLBP	Chronic Low Back Pain
PAM	Pneumatic Artificial Muscle
EMG	Electromyogram
IMU	Inertial Measurement Unit
RMS	Root Mean Square
FFT	Fast Fourier Transform
AR	Auto Regression
CoM	Center of Mass
PCB	Printed Circuit Board
GUI	Graphical User Interface
MVIC	Maximum Volitional Isometric
AUC	Area Under Curve

## References

[1] Wertheimer G., Mathieson S., Maher C.G., Lin C.-W.C., McLachlan A.J., Buchbinder R., Pearson S.-A., Underwood M. (2021). The Prevalence of Opioid Analgesic Use in People with Chronic Noncancer Pain: Systematic Review and Meta-Analysis of Observational Studies.

[2] Moses-Hampton M.K., Povieng B., Ghorayeb J.H., Zhang Y., Wu H. (2023). Chronic low back pain comorbidity count and its impact on exacerbating opioid and non-opioid prescribing behavior. Pain Pract..

[3] Deyo R.A., Dworkin S.F., Amtmann D., Andersson G., Borenstein D., Carragee E., Carrino J., Chou R., Cook K., Delitto A. (2015). Report of the NIH task force on research standards for chronic low back pain. Phys. Ther..

[4] Gatchel R.J., Reuben D.B., Dagenais S., Turk D.C., Chou R., Hershey A.D., Hicks G.E., Licciardone J.C., Horn S.D. (2018). Research Agenda for the Prevention of Pain and Its Impact: Report of the Work Group on the Prevention of Acute and Chronic Pain of the Federal Pain Research Strategy. J. Pain.

[5] Christie C., Baker C., Cooper R., Kennedy P.J., Madras B., Bondi P. (2017). The President’s Commission on Combating Drug Addiction and The Opioid Crisis Roster of Commissioners.

[6] Koes B.W., Van Tulder M.W., Thomas S. (2006). Diagnosis and Treatment of Low Back Pain.

[7] Deyo R.A., Weinstein J.N. (2001). Low Back Pain. N. Engl. J. Med..

[8] Anderson D.I., Lohse K.R., Lopes T.C.V., Williams A.M. (2021). Individual differences in motor skill learning: Past, present and future. Hum. Mov. Sci..

[9] Loeb G.E. (2021). Learning to use muscles. J. Hum. Kinet..

[10] Concha-Cisternas Y., Castro-Piñero J., Vásquez-Muñoz M., Molina-Márquez I., Vásquez-Gómez J., Guzmán-Muñoz E. (2024). Effects of neuromuscular training on postural balance and physical performance in older women: Randomized controlled trial. J. Funct. Morphol. Kinesiol..

[11] Antohe B.A., Panaet E.A. (2024). The Effects of Proprioceptive Exercises on Postural Control in Handball Players with Chronic Ankle Instability—A Non-Randomized Control Trial. Sports.

[12] Zemková E., Kováčiková Z. (2023). Sport-specific training induced adaptations in postural control and their relationship with athletic performance. Front. Hum. Neurosci..

[13] Hao Z., Cheng X., Jiang H., Yang J., Li Y., Lo W.L.A., Yu Q., Wang C. (2024). The associations between lumbar proprioception and postural control during and after calf vibration in people with and without chronic low back pain. Front. Bioeng. Biotechnol..

[14] Ghai S., Nilson F., Gustavsson J., Ghai I. (2024). Influence of compression garments on proprioception: A systematic review and meta-analysis. Ann. New York Acad. Sci..

[15] Chen Z., Tirosh O., Han J., Adams R., El-Ansary D., Pranata A. (2024). Lower Limb Proprioception in Low Back Pain and Its Relationship with Voluntary Postural Control. J. Mot. Behav..

[16] Nishi Y., Shigetoh H., Fujii R., Osumi M., Morioka S. (2021). Changes in trunk variability and stability of gait in patients with chronic low back pain: Impact of laboratory versus daily-living environments. J. Pain Res..

[17] Saein A.M., Kahrizi S., Boozari S. (2024). Effects of unstable load and unstable surface ontrunk muscles activation and postural control in healthy subjects. J. Biomech..

[18] Alshehri M.A., Alzahrani H., Hoorn W.V.D., Klyne D.M., Vette A.H., Hendershot B.D., Roberts B.W.R., Larivière C., Barbado D., Vera-Garcia F.J. (2024). Trunk Postural Control During Unstable Sitting Among Individuals with and Without Low Back Pain: A Systematic Review with an Individual Participant Data Meta-Analysis.

[19] Ippersiel P., Teoli A., Wideman T.H., Preuss R.A., Robbins S.M. (2022). The Relationship between Pain-Related Threat and Motor Behavior in Nonspecific Low Back Pain: A Systematic Review and Meta-Analysis. Phys. Ther..

[20] Cruise D.R., Chagdes J.R., Liddy J.J., Rietdyk S., Haddad J.M., Zelaznik H.N., Raman A. (2017). An active balance board system with real-time control of stiffness and time-delay to assess mechanisms of postural stability. J. Biomech..

[21] Allahverdi F., Korayem M.H. (2025). Design of a new balance rehabilitation cable robot focused on patient with cerebral palsy. J. Braz. Soc. Mech. Sci. Eng..

[22] Cinnera A.M., Ciancarelli I., Marrano S., Palagiano M., Federici E., Bisirri A., Iosa M., Paolucci S., Koch G., Morone G. (2024). Sensor-Based Balance Training with Exergaming Feedback in Subjects with Chronic Stroke: A Pilot Randomized Controlled Trial. Brain Sci..

[23] Fil-Balkan A., Salcı Y., Keklicek H., Armutlu K., Aksoy S., Kayıhan H., Elibol B. (2018). Sensorimotor integration training in Parkinson’s disease. Neurosciences.

[24] Porwal S., Rizvi M.R., Sharma A., Ahmad F., Alshahrani M.S., Raizah A., Shaik A.R., Seyam M.K., Miraj M., Alkhamis B.A. (2023). Enhancing Functional Ability in Chronic Nonspecific Lower Back Pain: The Impact of EMG-Guided Trunk Stabilization Exercises. Healthcare.

[25] Khan M.A., Shaik S., Tariq M.H., Kamal T. McKibben Pneumatic Artificial Muscle Robot Actuators—A Review. Proceedings of the 2023 International Conference on Robotics and Automation in Industry, ICRAI 2023.

[26] Krishnan S., Rani A.M.A., Kurappa L.G., Paramasivam S. (2022). Fabrication of Parallel Mechanism Actuated by Pneumatic Artificial Muscle for Rehabilitation Therapy. Lecture Notes in Electrical Engineering.

[27] Andrievsky B., Kuznetsov N.V., Kudryashova E.V., Kuznetsova O.A., Zaitceva I. (2023). Signal-parametric discrete-time adaptive controller for pneumatically actuated Stewart platform. Control. Eng. Pract..

[28] Ahern D.K., Follick M.J., Council J.R., Laser-Wolston N., Litchman H. (1988). Comparison of lumbar paravertebral EMG patterns in chronic low back pain patients and non-patient controls. Pain.

[29] Kohler J.M., Flanagan S.P., Whiting W.C. (2010). Muscle activation patterns while lifting stable and unstable loads on stable and unstable surfaces. J. Strength Cond. Res..

[30] Stegeman D., Hermens H. (2007). Standards for suface electromyography: The European project Surface EMG for non-invasive assessment of muscles (SENIAM). Ensch. Roessingh Res. Dev..

[31] Criswell E. (2010). Cram’s Introduction to Surface Electromyography.

[32] Silfies S.P., Mehta R., Smith S.S., Karduna A.R. (2009). Differences in Feedforward Trunk Muscle Activity in Subgroups of Patients with Mechanical Low Back Pain. Arch. Phys. Med. Rehabil..

[33] Zhong S., Gai Z., Yang Y., Zhao Y., Qi Y., Peng Y. (2022). A contraction length feedback method for the McKibben pneumatic artificial muscle. Sens. Actuators A Phys..

[34] Kalita B., Leonessa A., Dwivedy S.K. (2022). A Review on the Development of Pneumatic Artificial Muscle Actuators: Force Model and Application. Actuators.

[35] Kanno R., Watanabe S., Shimizu K., Shintake J. (2021). Self-Sensing McKibben Artificial Muscles Embedded with Dielectric Elastomer Sensor. IEEE Robot. Autom. Lett..

[36] Chou C.P., Hannaford B. (1996). Measurement and modeling of McKibben pneumatic artificial muscles. IEEE Trans. Robot. Autom..

[37] Jamil B., Oh N., Lee J.G., Lee H., Rodrigue H. (2024). A Review and Comparison of Linear Pneumatic Artificial Muscles. Korean Soc. Precis. Eng..

[38] Wang S., Pai Y.C., Bhatt T. (2022). Neuromuscular mechanisms of motor adaptation to repeated gait-slip perturbations in older adults. Sci. Rep..

[39] Zech A., Hübscher M., Vogt L., Banzer W., Hänsel F., Pfeifer K. (2010). Balance training for neuromuscular control and performance enhancement: A systematic review. J. Athl. Train..

[40] Kaplanoglu E. (2025). Athletic Balance Control Platform with Pneumatic Actuation.

